# Morin improves Bisphenol-A-induced toxicity in the rat testicular mitochondria and sperms

**DOI:** 10.5935/1518-0557.20220010

**Published:** 2023

**Authors:** Yousef Asadi-Fard, Maryam Zohour Soleimani, Mohamad Javad Khodayar, Layasadat Khorsandi, Maryam Shirani, Azin Samimi

**Affiliations:** 1 Student Research Committee, Ahvaz Jundishapur University of Medical Sciences, Ahvaz, Iran; 2 Department of Anatomical Sciences, Faculty of Medicine, Ahvaz Jundishapur University of Medical Sciences, Ahvaz, Iran; 3 Toxicology Research Center, Medical Basic Sciences Research Institute, Ahvaz Jundishapur University of Medical Sciences, Ahvaz, Iran; 4 Cellular and Molecular Research Center, Medical Basic Sciences Research Institute, Ahvaz Jundishapur University of Medical Sciences, Ahvaz, Iran; 5 Legal Medicine Research Center, Legal Medicine Organization, Ahvaz, Iran

**Keywords:** Morin, Bisphenol-A, sperm quality, mitochondria

## Abstract

**Objective:**

The present study aimed to examine the ameliorative effects of Morin (MRN) on Bisphenol-A (BPA)-induced oxidative stress in testicular mitochondria and sperm quality of rats.

**Methods:**

BPA and MRN (25, 50, and 100 µM) were given to the spermatozoa and testicular tissue mitochondria. The sperm quality, mitochondrial viability, and MMP (mitochondrial membrane potential) were examined. Superoxide dismutase, CAT (catalase), malondialdehyde, and reactive oxygen species (ROS) levels of rat testicular mitochondria were measured.

**Results:**

BPA raised mitochondrial oxidative stress biomarkers, whereas antioxidant acclivity and MMP were significantly lowered. BPA significantly lowered the normality, viability, and motility of the sperms. MRN dose-dependently lowered oxidative stress of the mitochondria, raised MMP, as well as improved the percentage of abnormality, motility, and viability of the sperms.

**Conclusions:**

These data demonstrated that MRN dose-dependently attenuated BPA-induced mitochondrial damage and improved sperm quality by preventing oxidative stress.

## INTRODUCTION

Bisphenol-A (BPA) is commonly used for producing clear and hard plastics for lining inside metal-based foods and beverage cans, sealants, toys, lenses, compact discs, etc… (Wazir & Mokbel, 2019; Vom Saal & Vandenberg, 2021). BPA through inhalation, ingestion, and dermal contact can penetrate into the human body. The exposure of individuals to BPA is associated with its environmental and food amounts (Ribeiro *et al*., 2017). BPA exists in breast milk, semen, plasma, and amniotic fluid (Suteau *et al*., 2020). BPA induces testicular damage and impairs the spermatogenesis of rats and other animals (Ullah *et al*., 2018; Anjum *et al*., 2011; Knez *et al*., 2014). BPA can impair mitochondrial function by lowering ATP, reducing mitochondrial mass, and altering the mitochondrial membrane integrity (Kaur *et al*., 2018). Mitochondrial dysfunction affects sperm generation and spermatozoa motility (Kamali Sangani *et al*., 2017). Additionally, BPA lowers antioxidant capacity and raises oxidative stress in testis (Chitra *et al*., 2003).

Current researches have focused on finding protective natural materials against male reproductive system dysfunctions. Recently, flavonoids have been extensively studied for ameliorating male reproductive damages (Farombi *et al*., 2012; Guvvala *et al*., 2017). Morin (MRN; a member of flavones) is plenty found in figs, sweet chestnuts, almonds, and other members of the *Moraceae* family. MRN was shown to have several beneficial impacts, including free radical scavenging, anti-tumorigenicity, anti-inflammatory, as well as DNA protection (Hussain *et al*., 2014; Meydanli *et al*., 2018). Recent studies have shown that MRN protects against sperm alterations and testicular oxidative stress induced by various chemicals in animals (Olayinka *et al*., 2018; Hussain *et al*., 2014).

The positive impacts of MRN against BSA-induced mitochondrial oxidative stress and impaired quality of rat sperms were the aim of the current study.

## MATERIALS AND METHODS

### Study design (Figure 1)

**Figure 1:**
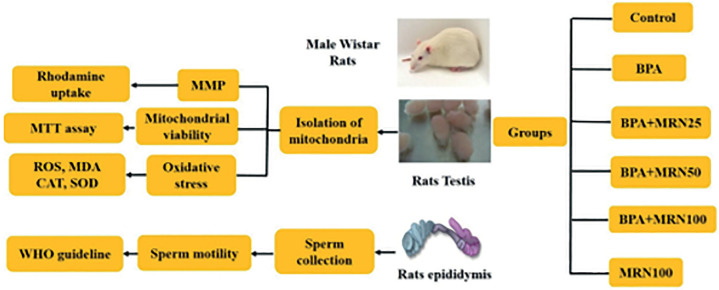
Study design of this work.

The sperms and mitochondria were obtained from 15 male Wistar rats (180-200 g). The Ethics Committee of Animal Research approved current work (REC: 1398-016). The sperms were obtained from the epididymis of the rats and categorized into the below groups:

Group 1. Treated with Ham’s F-10 media only for 4 hours (Control)

Group 2. Received BPA (0.8 µM) for 2 hours

Groups 3-5: Received 25, 50, and 100 µM MRN, respectively, for 2 hours before BPA.

Group 6 (MRN): Received 100 µM MRN for 4 hours

For each group 5×10^6^ sperm/ ml were used for treatment with MRN or BPA. Because the unexposed sperms died after 4 hours, a total time of 4 hours was set for treatment with MRN or BPA. MRN (Sigma) or BPA (Sigma) were dissolved in 0.1% DMSO and then were diluted in Ham’s F-10 media. BPA concentration was based on the MTT results (Table 1). The safety of DMSO was evaluated by MTT assay (Table 2).

**Table 1:** The impact of BPA on spermatozoa viability (Mean ± SD; n=6)

Treatment	1 hour	2 hours
0.0	100±0.00	100±0.00
0.1 µM	97.9±2.27	96.5±2.67
0.2 µM	91.8±4.53	85.4±4.13
0.4 µM	75.9±5.18	66.1±4.79
0.8 µM	62.8±4.33^*^	49.7±2.82^**^
1 µM	55.1±3.93^*^	34.6±3.17^**^

* *p*<0.05,

** *p*<0.01;

* comparison with control.

**Table 2:** Safety assessment of DMSO (0.1%) in the mitochondria and sperms

Parameters	Control	DMSO
Viability of mitochondria (%)	100±0.00	99.5±1.2
Viability of sperms (%)	100±0.00	99.2±1.4
Total sperm motility (%)	80.6±5.4	80.5±5.5
MMP (% of control)	100±0.00	100.03±1.1

Values are expressed as mean ± SD (n=6).

### Mitochondria isolation

The testicles of rats were dissected and minced in a buffer containing HEPES-KOH (5 mM), fat-free BSA (0.1%), EDTA (0.1 mM), EGTA (0.2 mM), and sucrose (250 mM), and then were homogenized. The mitochondrial fractions were centrifuged (at 4°C) 1 time for 10 minutes at 3,000·g and 3 times for 7 minutes at 10,000·g. The protein content was detected using the Bradford reagent (Invitrogen). An amount of 0.5 mg protein/ mL was exposed to BPA or MRN.

### MTT assay

Spermatozoa (5×10^6^ sperm/ml) or mitochondria were plated and exposed to MRN or BPA. MTT at a concentration of mg/ mL (Sigma, USA) was added to the treated subjects and incubated at 37°C for 1 hour. Then the media was discarded, and DMSO (100 µL) was added to the wells, and the absorbance was read at 570 nm.

### Sperm motility and morphology

In brief, 10 µL sperm suspension was placed in an analyzing semen chamber, and motility of at least 200 sperms was evaluated for each sample. Sperm motility was graded according to WHO guidelines to estimate the percentage of fast progressive (A), slow progressive (B), no progressive (C), and immotile sperm (D).

The sperm suspension was stained with 10%nigrosin and 1% eosin for morphological observations. For each group, 100 sperms with six replications were evaluated for abnormality estimation. The normal sperms had a hook-like shape acrosome with a straight neck and a free-end single tail. While the abnormal sperms had a smaller head, broken neck, branched tail, etc. The control and experimental samples were blindly analyzed with 3 co-workers.

### Determining antioxidant levels, MDA content, and ROS formation

The mitochondria of each group were placed into a micro-tube, and DCFH-DA (10 µM; Sigma) plus Hank’s buffered salt solution (100 µL) was added and incubated for 30 minutes. A spectrofluorometer (Ex: 490nm and Em: 570nm) was applied to detect ROS levels. Malondialdehyde (MDA), superoxide dismutase (SOD), and CAT (Catalase) levels were explored by an available commercial kit (ZellBio Company).

### Mitochondrial membrane potential (MMP) evaluation

The mitochondrial fractions were mixed with Rhodamine 123 (10µM) for 20 minutes. A spectrophotometer (LS50B, USA; emission: 535nm; excitation: 490nm) was applied for measuring the fluorescence.

### Statistical Analysis

Analysis of variance (one-way) in SPSS (version 21.0) followed by posthoc pairwise comparison was used in this study; *p*-values less than 0.05 were deemed significant.

## RESULTS

### MTT assay

**Figure 2:**
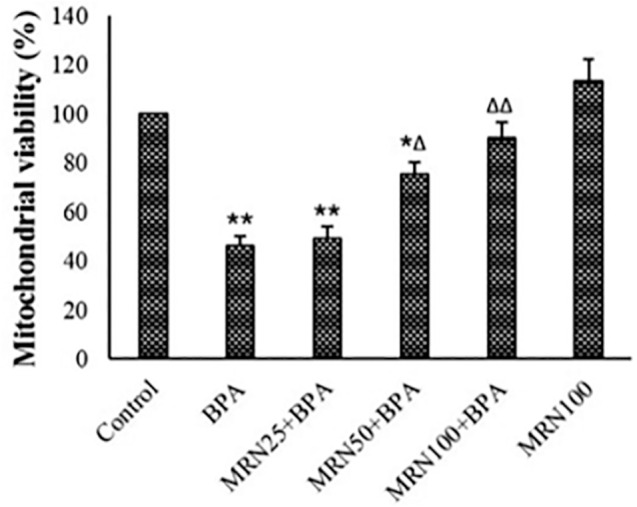
MTT assay results in different groups (Mean ± SD; n=6). *p<0.05, **p<0.01, ^∆^*p*<0.05, ^∆∆^*p*<0.01; *and ^∆^ showing comparisons against control and BPA groups.

**Figure 3:**
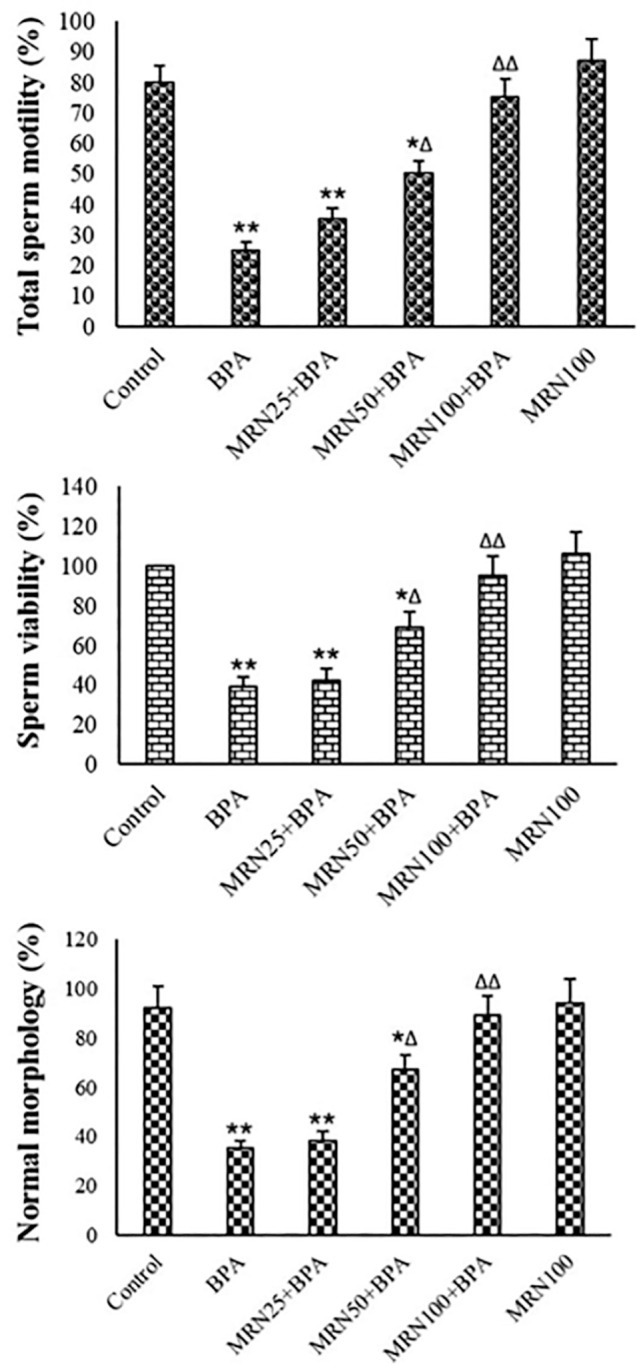
Sperm quality parameters in the various groups (Mean ± SD, n=6) *and ^∆^ show comparisons with control and BPA.

The survival rate of spermatozoids and testicular mitochondria was decreased considerably following exposure to BPA (*p*<0.01). MRN raised the viability of the sperms and mitochondria in the BPA treatment (Figures 2 and 3). MRN at a concentration of 100 µM could reverse the viability of sperms and mitochondria near to the control (*p*<0.01).

### Sperm quality

MRN slightly raised total sperm motility compared to controls. In the BPA treatment, the fast progressive percent (*p*<0.05) and total sperm motility (*p*<0.01) were significantly lowered, whereas the percentage of immotile spermatozoa was significantly raised (*p*<0.01). MRN at the concentrations of 50 and 100 µM could lower the percent of immotile sperm and raise total sperm motility after BPA treatment (Table 3 and Figure 3). The abnormality of sperms was significantly elevated in BPA-intoxicated sperms (*p*<0.01). Pretreatment with MRN could concentration-dependently attenuate the abnormality of the sperms in comparison to the BPA group.

**Table 3:** Velocity distribution of the sperms are presented (Mean ± SD; n=6)

Groups	Fast progressive	Slow progressive	No progressive	Immotile
Control	45.9±4.4	35.3±3.8	11.8±2.6	7.8±2.8
BSA	13.6±2.27^**^	12.9±2.14^*^	38.5±3.25^*^	35.2±2.7^**^
MRN25+BSA	23.5±3.2^**^	20.4±2.4	26.1±2.8^*^	29.6±3.3^**^
MRN50+BSA	26.6±3.7^*∆^	25.1±3.2	30.9±3.6	15.6±2.6^*∆∆^
MRN100+BSA	43.6±4.8 ^∆∆^	27.9±3.8^∆^	18.7±2.8^∆^	9.9±1.9 ^∆∆^
MRN100	46.4±6.3	36.3±3.5	11.3±2.7	6.2±1.32

^∆^ *p*<0.05, ^∆∆^*p*<0.01, ^∆∆∆^ *p*<0.001. ^*^and ^∆^ show comparisons with control and BPA

### Antioxidant levels, MDA content, and ROS formation

**Figure 4:**
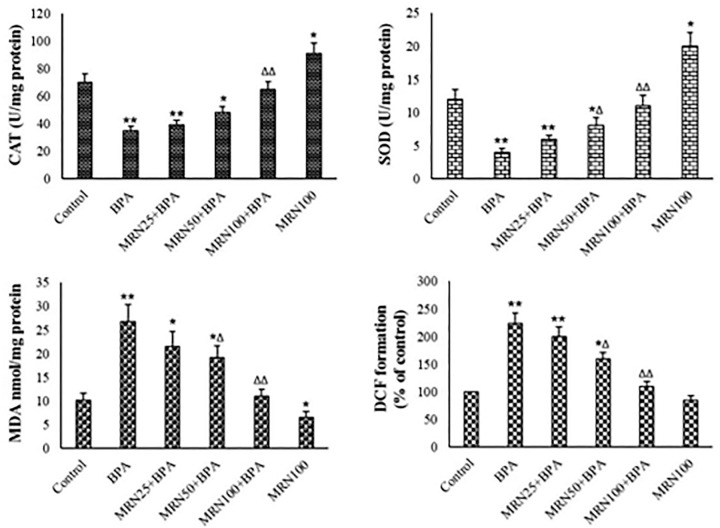
Mitochondrial levels of MDA, ROS, CAT, and SOD (Mean ± SD, n=6) *and ^∆^ show comparisons with control and BPA.

ROS and MDA levels were noticeably raised in the BPA group (*p*<0.01). MDA and ROS levels were lowered in the MRN-treated groups, compared with the control. MRN concentration-dependently lowered ROS generation in the BPA-exposed mitochondria (Figure 4). CAT and SOD activity were significantly lowered after treatment with BPA (*p*<0.01). In the MRN-treated mitochondria, the antioxidant activity was greater than the unexposed group. MRN concentration-dependently reversed the BPA-lowering antioxidant activity of the mitochondria (Figure 4).

### MMP Assay

**Figure 5:**
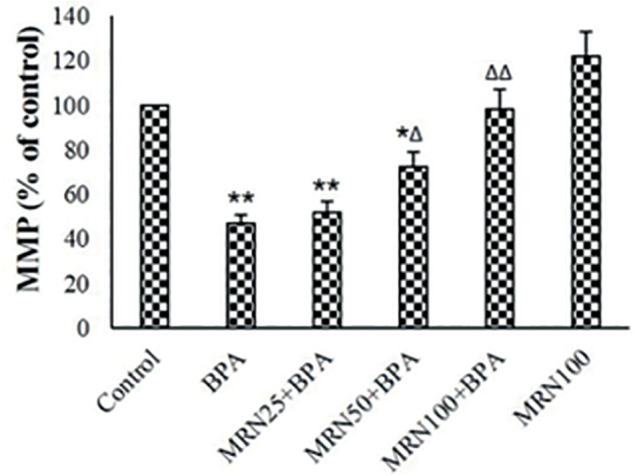
MMP assay in various groups (Mean ± SD, n=6) *and ^∆^ show comparisons with control and BPA.

In the BPA group, MMP significantly lowered compared with the control (*p*<0.01). MRN dose-dependently could raise the MMP of BPA-treated mitochondria (Figure 5). MRN at the concentration of 100 µM could reverse the MMP of BPA-exposed mitochondria near to the normal (*p*<0.01).

## DISCUSSION

The present study evidenced that BPA significantly reduced motility, normal morphology, and survival of the spermatozoa. In parallel with our finding, BPA lowered the quality of the sperm in several human and animal studies (Knez *et al*., 2014; Kotwicka *et al*., 2016). We found that MRN concentration-dependently enhanced viability, normal morphology rate, and fast progressive movement of the BPA-treated rat’s sperms. The amelioration effect of MRN against BPA-impaired sperm quality was similar to the previous reports (Khaki *et al*., 2010; Merwid-Ląd *et al*., 2014). MRN considerably reversed the altered sperm parameters in the procarbazine (an alkylating agent)-induced sperm toxicity in rats (Olayinka *et al*., 2018).

Arisha *et al*. (2019) have shown that silver nanoparticles reduce motility, viability, and concentration of sperms, disrupt the blood-testis barrier, and induce histological changes in rat testis. They have found that MRN can restore the reproductive alterations to their normal range. Hussein *et al*. (2019) reported that the administration of MRN with or without rutin led to a considerable decrease in the percentage of sperm abnormalities and alleviated the toxic effects of TiO_2_ nanoparticles. MRN may improve sperm survival by preventing cell death signaling. MRN could significantly change the ratio of the pro-apoptotic and anti-apoptotic molecules and effectively prevent apoptosis induced by silver nanoparticles in the testicular cells (Arisha *et al*., 2019). The MRN improved the motility of spermatozoa may be due to the beneficial impacts of MRN on the testicular mitochondrial (Ola *et al*., 2014).

The decreased sperm quality by the BPA was accompanied by enhancing oxidative stress. The BPA-raised ROS and MDA levels of the mitochondria were in line with other reports (Rahman *et al*., 2017; Kaur *et al*., 2018). The excessive generation of ROS attacks sperms and induces MDA production leading to lipid peroxidation (Ryu *et al*., 2017; Begum *et al*., 2018). MRN reversed MDA levels, ROS production, antioxidant biomarkers, and MMP in the BPA-exposed mitochondria. Therefore, MRN may protect spermatozoids via lowering ROS generation and lipid peroxidation. It has been documented that MRN protects mitochondrial from oxidative disorders in various pathological cases. MRN significantly enhanced antioxidant activity and lowered MDA level when co-administered with silver nanoparticles (Arisha *et al*., 2019). MRN could preserve antioxidant defense systems against rat testicular damages induced by bicalutamide (Olayinka *et al*., 2018). MRN could reverse SOD, CAT, GSH, and MDA levels in rat testicular toxicity induced by dutasteride-tamsulosin. In another study, MRN ameliorated TiO2-Nanoparticles-induced testicular and prostatic toxicity by activating antioxidant systems and reducing apoptosis (Hussein *et al*., 2019).

The BPA reducing MMP was similar to the previous studies (Wang *et al*., 2021; Mahdavinia *et al*., 2019; Barbonetti *et al*., 2016). MMP was positively correlated with total sperm numbers and progressive sperm motility (Zhang *et al*., 2016). The BPA reducing MMP was accompanied by enhancing mitochondrial oxidative stress, lowering spermatozoid motility, and attenuating sperm survival. BPA may cause mitochondrial damage by producing lipid peroxidation and impair spermatozoid function (del Hoyo *et al*., 2010). MRN concentration-dependently reversed the effects of BPA on MMP. Hence MRN may decrease ROS level by increasing MMP and consequently lowering germ cell loss. These results were in parallel to available documents that indicated the positive impacts of MRN on mitochondrial activity (Arisha *et al*., 2019). Ola *et al*. (2014) have reported that MMP and sperm quality of infertile individuals enhance in MRN-exposed samples.

## CONCLUSION

In summary, MRN could improve MMP and lowered mitochondrial oxidative stress. MRN could also effectively improve the normal morphology, survival, and motility of the rat sperms. MRN may ameliorate BPA-caused mitochondrial toxicity and rat sperm impairment by suppressing oxidative stress.
